# Saliva RNA Biomarkers of Gastrointestinal Dysfunction in Children With Autism and Neurodevelopmental Disorders: Potential Implications for Precision Medicine

**DOI:** 10.3389/fpsyt.2021.824933

**Published:** 2022-01-20

**Authors:** David Q. Beversdorf, Kristin Sohl, David Levitskiy, Priscilla Tennant, Robin P. Goin-Kochel, Rebecca C. Shaffer, Alexandra Confair, Frank A. Middleton, Steven D. Hicks

**Affiliations:** ^1^University of Missouri, Columbia, MO, United States; ^2^Quadrant Biosciences Inc., Syracuse, NY, United States; ^3^Department of Pediatrics, Baylor College of Medicine, Houston, TX, United States; ^4^Meyer Center for Developmental Pediatrics and Autism, Texas Children's Hospital, Houston, TX, United States; ^5^Cincinnati Children's Hospital, University of Cincinnati College of Medicine, Cincinnati, OH, United States; ^6^Department of Pediatrics, Penn State College of Medicine, Hershey, PA, United States; ^7^Department of Neuroscience and Physiology, The State University of New York, Upstate Medical University, Syracuse, NY, United States

**Keywords:** biomarkers, saliva, RNA, microRNA, autism (ASD), gastrointestinal

## Abstract

Gastrointestinal (GI) disorders are common in children with neurodevelopmental disorders such as autism spectrum disorder (ASD). A limited understanding of the biologic factors that predispose this population to GI disorders has prevented development of individualized therapies to address this important medical issue. The goal of the current study was to determine if elements of the salivary micro-transcriptome could provide insight into the biologic perturbations unique to children with ASD-related GI disturbance. This cohort study included 898 children (ages 18–73 months) with ASD, non-ASD developmental delay (DD), or typical development (TD). The saliva micro-transcriptome of each child was assessed with RNA-seq. Outputs were aligned to microbial and human databases. A Kruskal Wallis analysis of variance (ANOVA) was used to compare levels of 1821 micro-transcriptome features across neurodevelopmental status (ASD, DD, or TD) and GI presence or absence. An ANOVA was also used to compare micro-transcriptome levels among GI sub-groups (constipation, reflux, food intolerance, other GI condition, no GI condition), and to identify RNAs that differed among children taking three common GI medications (probiotics, reflux medication, or laxatives). Relationships between features identified in ANOVA testing were examined for associations with scores on the Autism Diagnostic Observation Schedule, 2nd Edition (ADOS-2) and the Vineland Adaptive Behavior Scales. GI disturbance rates were higher among children with ASD than peers with TD but were similar to those with DD. Five piwi-interacting RNAs and three microbial RNAs displayed an interaction between developmental status and GI disturbance. Fifty-seven salivary RNAs differed between GI sub-groups–with microRNA differences between food intolerance and reflux groups being most common. Twelve microRNAs displayed an effect of GI disturbance and showed association with GI medication uses and measures of behavior. These 12 microRNAs displayed enrichment for 13 physiologic pathways, including metabolism/digestion long-term depression, and neurobiology of addiction. This study identifies salivary micro-transcriptome features with differential expression among children with ASD-related GI disturbance. A subset of the RNAs displays relationships with treatment modality and are associated with autistic behaviors. The pathobiologic targets of the micro-transcriptome markers may serve as targets for individualized therapeutic interventions aimed at easing pain and behavioral difficulties seen in ASD-related GI disturbance.

## Introduction

Previous work has demonstrated that the salivary micro-transcriptome (non-coding RNA and microbial RNA) could be used to distinguish between children with autism spectrum disorder (ASD) (ages 2–6 years) and peers with typical development or developmental delay (1). These non-coding RNAs have regulatory roles in metabolism, cell differentiation and neuronal differentiation, by inhibiting gene expression (2). Elements of the micro-transcriptomes are up- or down-regulated by cells in response to the external environment (3), which suggests that they may have a dynamic relationship with other factors. Several non-coding RNA in saliva have demonstrated that their levels are associated with adaptive and autistic behaviors in children with ASD (4, 5), and are associated with socialization and autistic behaviors in young children with ASD (5). However, the relationship between non-coding RNA and comorbid conditions in ASD is not yet known.

Children with ASD appear to more frequently experience GI conditions than their neurotypical peers. Children with ASD have been reported to be diagnosed with a GI problem almost four times more often than children without ASD (6). The range of reported prevalence of GI symptoms is from 9 to 91% (7), likely a result of different methods of GI assessment. Constipation and diarrhea tend to be the most common GI diagnoses in ASD (6), with constipation the most common (8). Constipation can frequently be sufficiently severe to result in emergency department visits and hospital admissions among children with ASD (9).

Children with abdominal pain can also manifest difficult and distressing behaviors such as irritability, social withdrawal, stereotypy, and hyperactivity, as well as aggression and self-injurious behaviors (7, 10, 11). Associated comorbid conditions can include seizures, anxiety, depressed mood, attention-deficit/hyperactivity disorder, oppositional defiant disorder, sleep problems, as well as other problem behaviors (12–16). Problem behavior may, itself, sometimes be an indicator of GI distress in ASD, particularly among individuals with ASD with limited language (7). Younger individuals with ASD and GI disturbances display more externalizing behaviors such as aggression, and older individuals with ASD display more internalizing symptoms such as anxiety and depression (16). Stress reactivity as well as anxiety and autonomic arousal are also critically interrelated to severity of GI symptoms in ASD (17, 18). Many ASD patients with GI disturbances, such as constipation, are less likely to respond to first line therapies, such as stool softeners (19). Therefore, a better understanding of the biologic processes driving GI disturbances in patients with ASD might provide mechanistic insights toward better treatments for GI pain and related behaviors.

Heterogeneity across the autism spectrum has led to failures in many of the early clinical trials attempting to target core features of ASD (20). While micro-transcriptomes appear to distinguish ASD patients from neurotypical controls (21), they may also have particular value in helping to distinguish specific subtypes of ASD, which might impact treatment. Because of the significant adverse effects of GI disorders in ASD (6, 9), as well as their interrelationships with behavioral disturbances (12–16), we sought to examine differential micro-transcriptome expression in ASD patients with and without GI disturbances, as well as how this interrelates with behaviors. A greater understanding of the downstream targets of the differentially expressed micro-transcriptomes may help guide future personalized medicine approaches to treatment of GI disturbances in ASD.

## Methods

### Ethics

The study was approved by the Western Institutional Review Board (IRB #20180172). Written consent was obtained for all participants, and written informed assent was documented for those capable of assent.

### Participants

This case control study included a total of 898 children, ages 18–73 months, who were recruited from outpatient pediatric clinics affiliated with seven academic medical centers: Penn State University (*n* = 312), State University of New York (SUNY) Upstate Medical University (*n* = 335), Missouri University (*n* = 108), Cincinnati Children's Hospital (*n* = 45), Texas Children's Hospital (*n* = 54), University of California Irvine (*n* = 15); and University of Iowa (*n* = 29). Participants were divided into three groups based on neurodevelopmental status: autism spectrum disorder (ASD; *n* = 503), non-ASD developmental delay (DD; *n* = 205), and typical development (TD; *n* = 190). ASD status was determined by trained clinicians using DSM-5 criteria in association with standardized assessment tools (i.e., the Autism Diagnostic Observation Schedule, 2nd Edition; ADOS-2). DD participants included children referred for initial ASD assessment who did not meet diagnostic criteria, as well as children with negative ASD screening tools who required early intervention services for delays in gross motor, fine motor, language, or cognitive development (as reported by parental survey and confirmed through review of the medical record). TD participants included children recruited at the time of their annual well child visits who did not exhibit developmental delays on standard developmental surveillance tools (e.g., Survey of Wellbeing in Young Children, Parents' Evaluation of Developmental Status–Developmental Milestones, Modified Checklist for Autism in Toddlers Revised). Participants were further subdivided by presence (*n* = 184) or absence (*n* = 714) of gastrointestinal (GI) disturbance, based on: (2) parent report of (a) constipation (*n* = 84); (b) reflux (*n* = 46); (c) chronic diarrhea or abdominal pain (*n* = 22); (d) food intolerance (*n* = 45); (e) cyclic vomiting/dysphagia (*n* = 3); or (f) eosinophilic esophagitis (*n* = 7). Note that total numbers of specific conditions exceed the number of participants with a GI disturbance because 23 participants reported more than one type of GI disturbance. Exclusionary criteria for all participants included Ward of the State, g-tube dependence, active periodontal infection or acute upper respiratory illness.

### Measures

#### Participant Characteristics

The ADOS-2 was administered by trained raters to children with ASD (*n* = 409), and children with DD (*n* = 121) in whom ASD was suspected. The Vineland Adaptive Behavioral Scales 3rd Edition (VABS-III) was used to measure adaptive behavior, communication, and social interaction for all participants. Additionally, medical and demographic information including sex, age, race, ethnicity, medical conditions, and medications, was collected through parental surveys and affirmed via review of the electronic health record where available.

#### Saliva Collection and Processing

Saliva was obtained from all participants in a non-fasting state via swab, targeting the base of the tongue and between the gums and buccal mucosa as locations for the collection using an Oracollect RNA swab (DNA Genotek, Ottawa, Canada). Nucleic acid extraction was performed using the Qiagen miRNeasy Microkit (Cat. No. 217084), a QIAzol based purification method. The RNA sequencing process included using an Illumina TrueSeq Small RNA Prep protocol for library construction, followed by sequencing on an Illumina flow-cell and a NextSeq 500 instrument (Illumina; San Diego, CA, United States). Sequencing outputs were a binary base call (BCL) sequence file per sample, which was then converted to a FASTQ file, a text-based format that includes detected bases and associated quality scores (i.e., confidence in correct detection). Alignment and quantification of known RNA sequences for each collected specimen was done using the Bowtie1 aligner (22) to the following reference databases: miRBase v22 (23), piRBase v1 (24), RefSeq v90 (25), and hg38. Quantification of the detected sequences yielded counts of known human micro-ribonucleic acids (miRNAs), long non-coding transcripts (small nucleolar RNAs), and piwi-interacting RNA (piRNAs). To determine microbial RNAs present in the sample, the leftover sequences that did not align to hg38 were aligned to the NCBI microbial database using k-SLAM, an efficient aligner used in metagenomic data. Aligned sequences were then assigned to microbial genes, which were quantified to a microbial identity (e.g., genus, species, strain). Prior to analysis and count normalization, low count RNAs were removed from further analysis so that only reliably expressed RNAs were interrogated. Tabulated counts of each RNA were compared to the total counts detected in that RNA category, and RNAs that did not account for at least 0.01% of the total were dropped. Following abundance filtering, the remaining RNAs were quantile normalized and mean-center scaled.

### Statistics

The primary goals of the study were to: (2) identify human and microbial RNA levels in saliva that were associated with GI disturbance; (3) investigate whether these relationships were impacted by child developmental status; and (4) determine if specific RNA “biomarkers” displayed unique expression patterns in particular GI disturbances (e.g., constipation) or with particular treatments (e.g., probiotics). A two-way analysis of variance (ANOVA) was used to compare levels of 1821 RNA among the 898 participants based upon two factors: (2) neurodevelopmental status (ASD, DD, or TD); and (3) GI status (presence or absence of any GI condition). Interactions between neurodevelopmental status and GI status were reported. A one-way Kruskal Wallis rank sum test was used to identify RNAs that differed among GI sub-groups (constipation, reflux, food intolerance, other GI condition, no GI condition), and to identify RNAs that differed among those taking three common GI medications (probiotics, reflux medication, or laxatives). Finally, given the potential associations between underlying GI disturbance and child behaviors, relationships between RNAs identified in ANOVA testing, as well as the all one-way Kruskal Wallis testing were examined for associations with scores on the ADOS-2 and Vineland using Spearman Rank Testing. Benjamini Hochberg multiple testing correction was applied to all analyses. Functional analysis of candidate miRNAs (features displaying an interaction effect between neurodevelopmental status and GI disturbance, as well as relationships with treatment or behavior) was performed in DIANA miRPATH software v3.0 (26). The microT-cds algorithm (0.95 microT Threshold) was used to identify pathways over-represented by putative messenger RNA targets by Fisher Exact Test with Benjamini Hochberg multiple testing correction. Additionally, the rates of different demographic features were tested in the population. To test for differences in age by diagnosis (ASD, DD, TD) or presence of a GI disturbance, a one-way ANOVA was used. To test for differences in rates of sex, race, ethnicity, GI disturbance, constipation, reflux, food intolerance, chronic abdominal pain, diarrhea, or eosinophilic esophagitis, a chi-squared test was used yielding the chi-squared test statistic (x) and the associated *p*-value (p).

## Results

### Participants

Participating children had an average age of 44 (±16) months. They were mostly Caucasian (691/898, 76%), non-Hispanic (799/898, 89%), and male (663/898, 73%) (Table 1).

**Table 1:** Participant characteristics.

	**All (***n*** = 898)**	**ASD (***n*** = 503)**	**DD (***n*** = 205)**	**TD (***n*** = 190)**
Age, months (SD)	44 (16)	44 (16)	43 (15)	47 (18)
Sex, # male (%)	663 (73)	399 (79)	147 (71)	117 (61)*
White, # (%)	691 (76)	373 (74)	160 (78)	158 (83)*
Black, # (%)	115 (12)	72 (14)	29 (14)	14 (7)*
Asian, # (%)	22 (2)	17 (3)	3 (1)	2 (1)
Other race, # (%)	100 (11)	55 (10)	25 (12)	20 (10)
Hispanic, # (%)	99 (11)	70 (13)	18 (8)	11 (5)*
GI disturbance, # (%)	184 (20)	114 (22)	50 (24)	20 (10)*
Constipation, # (%)	84 (9)	57 (11)	20 (9)	7 (3)*
Reflux, # (%)	46 (5)	35 (6)	10 (4)	1 (0.5)*
Food intolerance, # (%)	45 (5)	23 (4)	11 (5)	11 (5)
Chronic abdominal pain or diarrhea, # (%)	22 (2)	16 (3)	6 (2)	1 (0.5)
Cyclic vomiting or dysphagia, # (%)	3 (0.3)	0 (0)	3 (1)*	0 (0)
Eosinophilic esophagitis, # (%)	7 (0.7)	4 (7)	3 (1)	0 (0)

The number of participants with specific GI disturbances exceeds the total number of participants with any GI disturbance (n = 184) because 22 participants reported more than one GI disturbance. *Denotes significant difference (p < 0.05) compared with ASD group on chi-square testing.

There were more males in the children with ASD (399/503, 79%) than in the children with TD (117/190, 61%) (*p* = 0.00000177, *x* = 22.83). There were fewer children with ASD who reported White race (373/503, 74%) than children with TD (158/190, 83%) (*p* = 0.0125, *x* = 6.24). More children with ASD reported Black race (72/503, 14%) and Hispanic ethnicity (70/503, 13%), compared to children with TD (14/190, 7%; 11/190, 5%, respectively) (*p* = 0.0134, *x* = 6.12; *p* = 0.00297, *x* = 8.82, respectively). There was no difference between ASD/DD/TD groups in age (*p* = 0.0588). There were limited differences between ASD/DD groups in sex (*p* = 0.029, *x* = 4.79), and no differences in reported White race (*p* = 0.276, *x* = 1.19), Black race (*p* = 0.954, *x* = 0.00335), or ethnicity (*p* = 0.0603, *x* = 3.53).

A higher proportion of children with ASD reported GI disturbance (114/503, 22%) than children with TD (20/190, 10%) (*p* = 0.000307, *x* = 13.03). Among children with ASD, reported rates of constipation (57/503, 11%) and reflux (35/503, 6%) were higher than reported rates among children with TD (7/190, 3%; and 1/190, 0.5%, respectively) (*p* = 0.00416, *x* = 8.21; *p* = 0.0025, *x* = 9.14, respectively). There were no differences between children with ASD and children with DD in rates of constipation (*p* = 0.78, *x* = 0.077), reflux (*p* = 0.46, *x* = 0.54), food intolerance (*p* = 0.86, *x* = 0.031), chronic abdominal pain (*p* = 0.77, *x* = 0.085), diarrhea (*p* = 0.797, *x* = 0.066), or eosinophilic esophagitis (*p* = 0.415, *x* = 0.664). There was no difference in age (*p* = 0.205), sex (*p* = 0.87, *x* = 0.0255), White race (*p* = 0.909, *x* = 0.0132), Black race (*p* = 0.395, *x* = 0.723), and limited differences in ethnicity (*p* = 0.041, *x* = 4.15) between children with/without GI disturbance.

### Impact of GI Disturbance on Saliva RNAs

**Figure 1:**
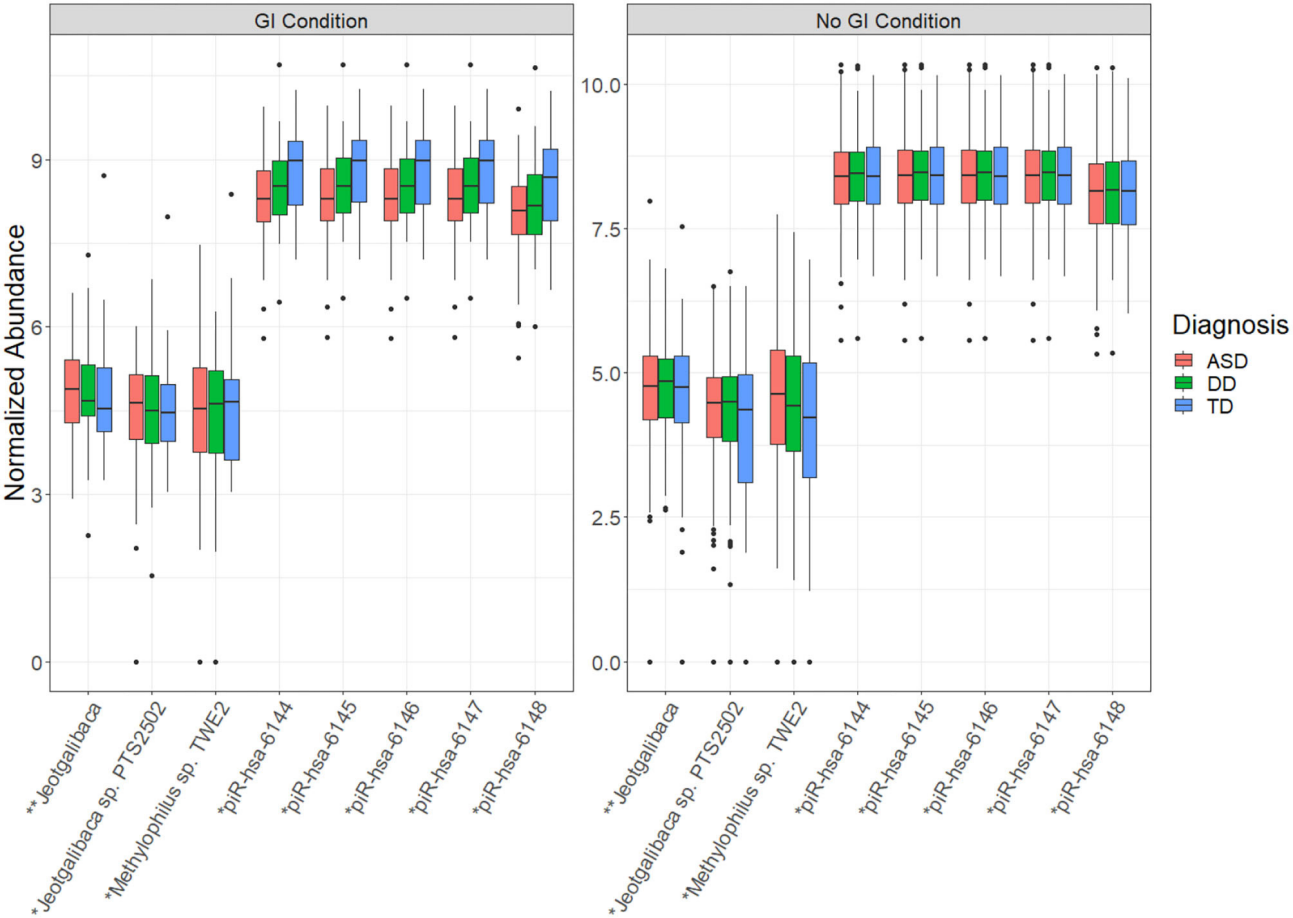
Transcript abundance displaying significant interaction between neurodevelopmental status and presence/absence of GI condition. The whisker box plots represent normalized abundance of microbial RNAs and piRNAs that displayed a significant interaction between neurodevelopmental status (ASD/DD/TD) and presence/absence of any GI condition. These features may provide insight into the unique biology underlying the heightened prevalence of GI conditions in children with ASD. Significance levels: *p*-val < 0.001 (*), *p*-val < 0.0001 (**). For all features shown, the adjusted *p*-value (FDR) <0.05.

Among the 1821 RNA features interrogated, 28 displayed a significant difference (adj *p* < 0.05) between children with/without GI disturbance (Table 2A). These RNA features included four mature miRNAs and 24 small non-coding RNAs, but no microbial RNAs. There were eight RNA features that displayed a significant interaction effect between neurodevelopmental status (ASD/DD/TD) and presence/absence of GI condition (Table 2B). These RNA features included five piRNAs and three microbial RNAs (Figure 1). The piRNAs tended to display similar saliva levels across ASD/DD/TD groups without GI disturbance, but were lower among children with ASD and GI disturbance, relative to peers with TD and GI disturbance.

**Table 2A:** Transcripts with significant differences between children with and without GI disturbances.

**Transcript**	***P*** **-value**	**Critical value**
hsa-miR-224-5p	0.000493513	3.52E-04
hsa-miR-27a-3p	0.000602833	7.04E-04
hsa-miR-27b-3p	0.000609137	0.001056338
hsa-miR-151a-5p	0.001025943	0.001408451
NR_029493.1	0.000202054	3.79E-04
NR_002579.1	0.001114037	7.58E-04
NR_000007.1	0.0019312	0.001136364
NR_003689.1	0.001943995	0.001515152
NR_145802.2	0.002630256	0.001893939
NR_002439.1	0.002949461	0.002272727
NR_003688.1	0.003909225	0.002651515
NR_002744.1	0.005420832	0.003030303
NR_023363.1	0.005674379	0.003409091
NR_023364.1	0.005674379	0.003787879
NR_023365.1	0.005674379	0.004166667
NR_023366.1	0.005674379	0.004545455
NR_023367.1	0.005674379	0.004924242
NR_023368.1	0.005674379	0.00530303
NR_023369.1	0.005674379	0.005681818
NR_023370.1	0.005674379	0.006060606
NR_023372.1	0.005674379	0.006439394
NR_023373.1	0.005674379	0.006818182
NR_023374.1	0.005674379	0.00719697
NR_023375.1	0.005674379	0.007575758
NR_023376.1	0.005674379	0.007954545
NR_023377.1	0.005674379	0.008333333
NR_023378.1	0.005674379	0.008712121
NR_023379.1	0.005674379	0.009090909

**Table 2B:** Transcripts displaying a significant interaction effect between neurodevelopmental status and the presence/absence of GI condition.

**Transcript name**	***P*** **-value**	**Critical value**
piR-hsa-6148	0.000493282	2.89E-04
piR-hsa-6145	0.000565113	5.78E-04
piR-hsa-6147	0.000566969	8.67E-04
piR-hsa-6146	0.000571064	0.001156069
piR-hsa-6144	0.000591411	0.001445087
Jeotgalibaca	3.31E-05	3.70E-04
Methylophilus sp. TWE2	0.000209947	2.20E-04
Jeotgalibaca sp. PTS2502	0.000398509	4.41E-04

### Differences in Saliva RNA Levels Among GI Phenotypes

**Figure 2:**
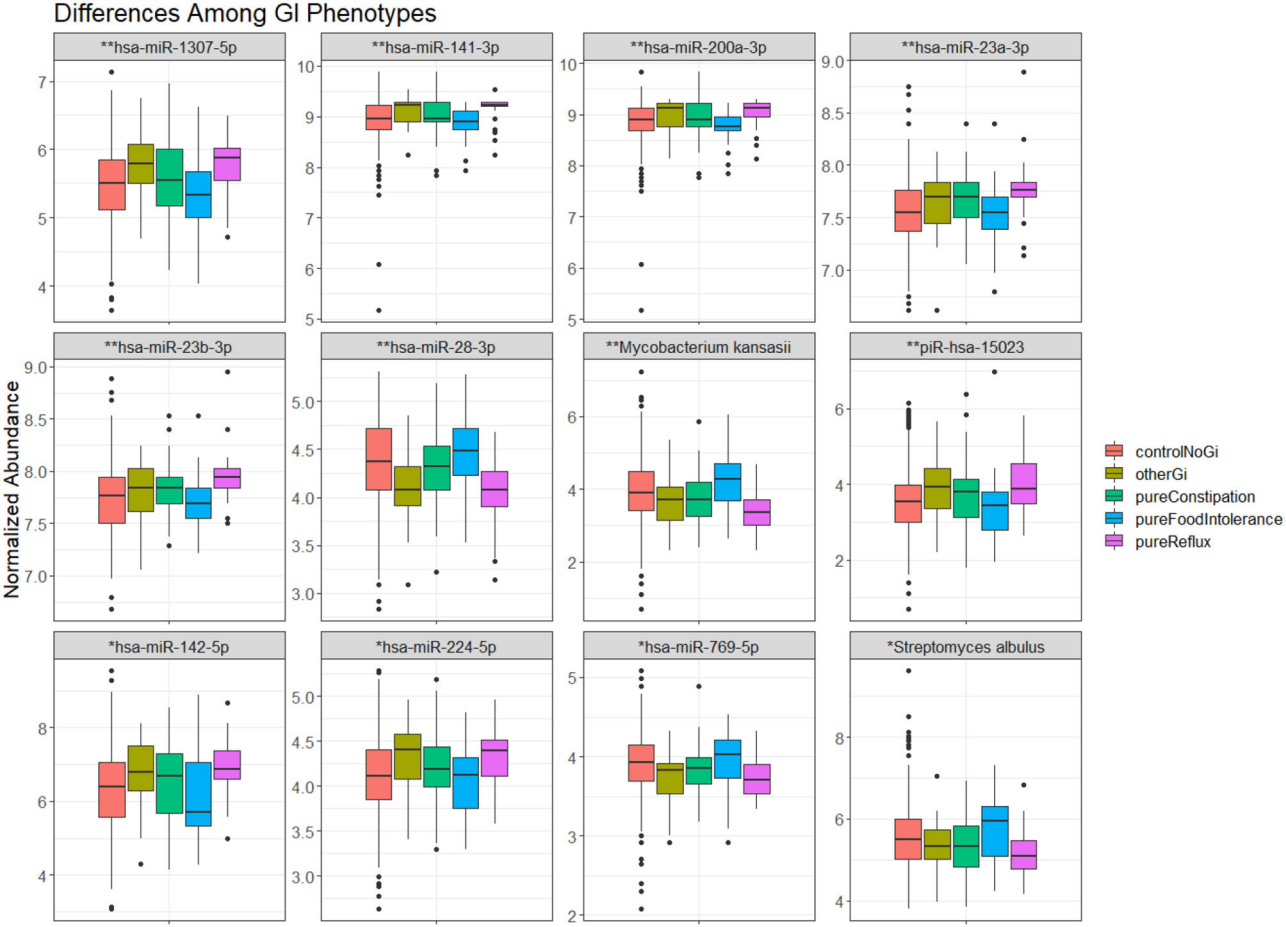
The most significant (12) transcripts differing between GI phenotypes. The whisker box plots represent normalized abundance of miRNAs and microbial RNAs that displayed a significant difference between children without a GI condition (orange), children with constipation (green), food intolerance (blue), reflux (pink), or another GI condition (brown). Significance levels–*p*-val <0.001 (*), *p*-val <0.0001 (**).

There were 57 RNA features that differed between GI phenotypes (Table 3). These RNA features included 12 microbial RNAs, three piRNAs, and 42 miRNAs. The largest differences tended to occur in miRNA levels, and were most common between children with reflux and food intolerance (Figure 2).

**Table 3:** Transcripts significantly different between GI phenotypes.

**Transcript**	***P*** **-value**	**Critical value**
hsa-miR-28-3p	4.80E-06	0.001056
hsa-miR-1307-5p	8.54E-06	0.002113
hsa-miR-200a-3p	3.33E-05	0.003169
hsa-miR-141-3p	3.77E-05	0.004225
hsa-miR-23a-3p	4.43E-05	0.005282
hsa-miR-23b-3p	5.02E-05	0.006338
hsa-miR-142-5p	1.31E-04	0.007394
hsa-miR-224-5p	1.70E-04	0.008451
hsa-miR-769-5p	3.36E-04	0.009507
hsa-miR-148a-5p	3.74E-04	0.010563
hsa-let-7b-5p	7.27E-04	0.01162
hsa-miR-27a-3p	7.42E-04	0.012676
hsa-let-7a-5p	8.19E-04	0.013732
hsa-let-7c-5p	0.001301	0.014789
hsa-miR-532-5p	0.001603	0.015845
hsa-miR-192-5p	0.002351	0.016901
hsa-miR-186-5p	0.002528	0.017958
hsa-miR-106b-3p	0.003164	0.019014
hsa-miR-200a-5p	0.003643	0.02007
hsa-miR-151a-3p	0.003758	0.021127
hsa-let-7e-5p	0.004643	0.022183
hsa-miR-181a-5p	0.005052	0.023239
hsa-miR-25-3p	0.006292	0.024296
hsa-miR-29c-3p	0.006425	0.025352
hsa-miR-10b-5p	0.00701	0.026408
hsa-miR-22-3p	0.007061	0.027465
hsa-miR-501-3p	0.008192	0.028521
hsa-miR-24-3p	0.009503	0.029577
hsa-miR-27b-3p	0.011937	0.030634
hsa-miR-182-5p	0.0138	0.03169
hsa-miR-3074-5p	0.016482	0.032746
hsa-miR-26b-5p	0.021226	0.033803
hsa-let-7f-5p	0.023545	0.034859
hsa-miR-125b-5p	0.02362	0.035915
hsa-miR-375-3p	0.02457	0.036972
hsa-miR-374a-5p	0.024781	0.038028
hsa-miR-92a-3p	0.029143	0.039085
hsa-miR-148a-3p	0.029199	0.040141
hsa-miR-425-5p	0.029741	0.041197
hsa-miR-222-3p	0.030985	0.042254
hsa-miR-30e-5p	0.035843	0.04331
hsa-miR-30b-5p	0.038612	0.044366
piR-hsa-15023	9.83E-05	8.67E-04
piR-hsa-28405	5.27E-04	0.001734
piR-hsa-17560	0.002069	0.002601
Mycobacterium kansasii	2.13E-06	6.61E-04
Streptomyces albulus	3.08E-04	0.001322
Staphylococcus simulans	8.04E-04	0.001982
Actinomyces radicidentis	0.001163	0.002643
Sneathia amnii	0.001933	0.003304
Lysinibacillus sphaericus	0.002427	0.003965
Candidatus Azobacteroides	0.002545	0.004626
pseudotrichonymphae		
Cellulomonas gilvus	0.00321	0.005286
Actinobacillus succinogenes	0.003283	0.005947
Capnocytophaga haemolytica	0.00522	0.006608
Corynebacterium singulare	0.006309	0.007269
Streptococcus dysgalactiae	0.007484	0.00793

### Effect of Medications on Saliva RNAs

Levels of 65 RNA features differed among children with GI disturbance on probiotics (*n* = 22) and children with GI disturbance not taking probiotics (*n* = 162) (Supplementary Table 1). These RNA features included 37 miRNAs, 75 piRNAs, one small non-coding RNA, and one microbial RNA. Levels of 53 RNA features differed among children with GI disturbance on laxatives (i.e., polyethylene glycol; *n* = 39) and children with GI disturbance not taking laxatives (*n* = 145). These RNA features included 15 microbial RNAs, seven small non-coding RNAs, four piRNAs, 27 miRNAs.

### Relationship of GI-Related Saliva RNAs and Child Behavior

There were 224 RNA features that displayed a significant relationship (adj *p* < 0.05) with at least one measure of child behavior on the VABS or the ADOS-2 (Supplementary Table 2). These RNA features included 47 miRNAs, 69 piRNAs, 16 small non-coding RNAs, and 92 microbial RNAs. The largest number of relationships were observed between RNA features and Vineland Communication Scores (*n* = 132).

### Functional Implications of Saliva miRNA Candidates

There were 12 salivary miRNAs that displayed relationships with GI disturbance, GI medications, and child behavior (miR-1307-5p, miR-141-3p, miR-142-5p, miR-148a-5p, miR-186-5p, miR-200a-3p, miR-200a-5p, miR-23a-3p, miR-23b-3p, miR-28-3p, miR-532-5p, and miR-769-5p). Together, these 12 miRNAs display enrichment for 13 KEGG pathways, including several implicated in metabolism/digestion (steroid biosynthesis, porphyrin metabolism, drug metabolism, ascorbate metabolism, lysine degradation, calcium reabsorption, and thyroid hormone signaling), and neurobiology (long-term depression, morphine addiction) (Table 4).

**Table 4:** KEGG pathway enrichment.

**KEGG pathway**	***p*** **-value**	**#Genes**	**#miRNAs**
Steroid hormone biosynthesis	3.04E-12	7	2
Hippo signaling pathway	5.03E-07	15	9
Gap junction	0.001139	10	7
Porphyrin and chlorophyll metabolism	0.005067	9	3
Endocrine and other factor-regulated calcium reabsorption	0.005067	6	7
Drug metabolism–cytochrome P450	0.007162	8	4
Glycosphingolipid biosynthesis–lacto and neolacto series	0.009386	4	5
Ascorbate and aldarate metabolism	0.016999	7	2
Lysine degradation	0.025194	5	7
Proteoglycans in cancer	0.026807	22	9
Thyroid hormone signaling pathway	0.027249	10	7
Long-term depression	0.030698	6	4
Morphine addiction	0.034341	9	7

## Discussion

In this cohort study of 898 children, rates of GI disturbance were higher among children with ASD than peers with TD, as expected (6), but were similar to those with DD. There were five piRNAs and three microbial RNAs in saliva that displayed an interaction between developmental status and GI disturbance (Figure 1). These features may serve as biomarkers for the unique pathophysiology leading to elevated GI disturbance in children with ASD. There were many salivary RNAs whose levels differed between GI disturbance phenotypes–with miRNA differences between food intolerance and reflux groups being most common. Levels of 12 salivary miRNAs that displayed an effect of GI disturbance were also associated with GI medications and measures of child behavior (miR-1307-5p, miR-141-3p, miR-142-5p, miR-148a-5p, miR-186-5p, miR-200a-3p, miR-200a-5p, miR-23a-3p, miR-23b-3p, miR-28-3p, miR-532-5p, and miR-769-5p).

The 12 salivary miRNAs that displayed relationships with GI disturbance, GI medications, and child behavior may serve as examples of biologic targets for personalized diagnostic and therapeutic approaches in children with ASD-related GI disturbance. Putative targets of these 12 miRNAs include transcripts that code for key regulators of both metabolism (e.g., steroid biosynthesis, porphyrin metabolism, ascorbate metabolism, calcium reabsorption, thyroid hormone signaling) and neurobiology (e.g., long-term depression). Intriguingly, exogenous steroids, porphyria, hypercalcemia, hypothyroidism, and depression are all associated with constipation and/or abdominal pain. It is possible that the 12 miRNAs contribute to sub-clinical perturbations in these physiologic pathways, in so-much-as they lead to GI pain without causing other overt clinical symptoms. For example, rodent models have demonstrated that restoration of miR-148a expression in the lower GI tract may reduce colitis (27), while elevations in miR-200a may lead to irritable bowel-like symptoms through inhibition of serotonin and cannabinoid transporters (28).

**Figure 3:**
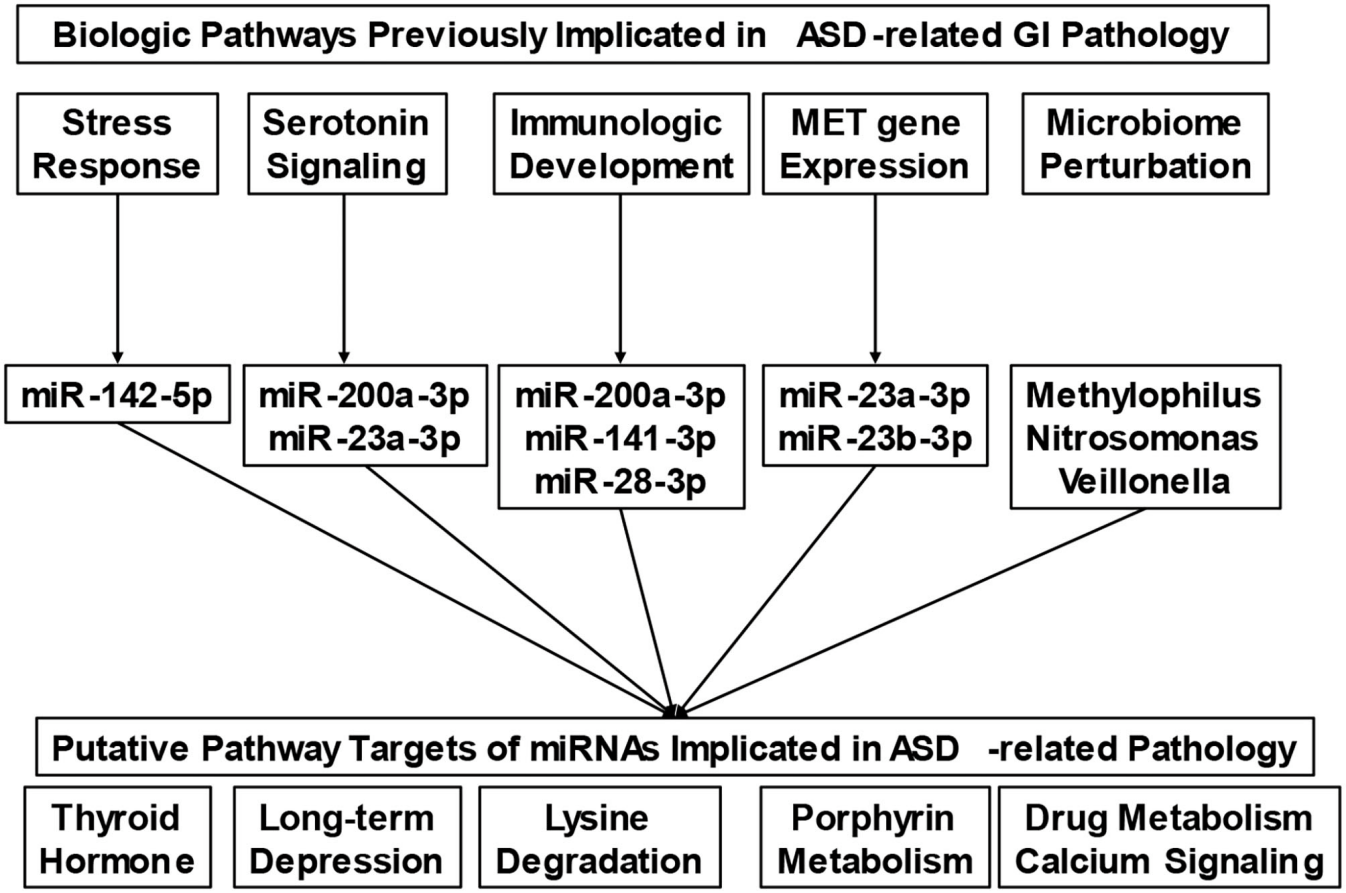
Putative micro-transcriptome links between biologic mechanisms and physiologic pathways involved in gastrointestinal pathophysiology. The conceptual diagram connects biologic pathways that have been previously implicated in Autism Spectrum Disorder (ASD)-related gastrointestinal (GI) pathology with putative micro-transcriptome features (i.e., microRNAs and microbes). Micro-transcriptome features were included based on their associations with the biologic pathways of interest in the existing literature. Collectively, the microRNAs display target enrichment for several clinical findings (i.e., thyroid hormone signaling, long-term depression, etc.), that are known contributors to GI disturbance.

Our understanding of the nature of GI disturbances in ASD is only beginning to emerge. Numerous pathways, including autonomic arousal (17, 18), serotonin dysregulation (29), and perturbations in gene expression (30) have all been implicated in this process. The micro-transcriptome features identified in this study provide a single mechanism through which each of these pathways may converge (Figure 3). For example, recent research has found that stress reactivity, anxiety, and autonomic arousal are interrelated with the severity of lower GI symptoms in ASD (17, 18). One miRNA identified in this study, miR-142-5p, has been previously implicated in anxious behavior following prolonged stress (31). Whole blood serotonin levels have also been associated with lower GI symptoms in ASD (29). Here, we identify one miRNA (miR-23a-3p) implicated in ASD-related GI pathology, which has previously been shown to change in depressed patients treated with selective serotonin reuptake inhibitors (32). Specific genes, in particular polymorphisms of the Mesenchymal Epithelial Transition (*MET*) receptor kinase gene, are also associated with GI symptoms in ASD (30). The MET receptor has been shown to modulate miRNA expression (33), and the MET transcript is a putative target of two miRNAs in the present study (miR-23a-3p, miR-23b-3p).

Immunological factors have also been found to be associated with GI symptoms as well as behavior in ASD (34, 35). The unique immunologic patterns associated with ASD may contribute to specific alterations in the microbiome profile that have been reported in children with ASD and GI symptoms (36). Evidence of this nature has even led to efforts at interventions based on the microbiome in ASD (37, 38). In the present study, we identify several miRNAs that are implicated in immune development. For example, miR-28 has been shown to modulate T-cell differentiation and cytokine expression (39), while miR-200a-3p and miR-141-3p have been found to work together to modulate differentiation of interleukin-producing T-helper cells (40). We found minimal overlap between the specific microbes identified in this study, and those of previous GI microbiome studies (36). This may be because the current investigation examines microbial RNA levels in saliva, as opposed to the more traditional 16S approach, using stool samples.

As further research begins to reveal a clearer understanding of the pathways implicated in ASD patients with GI symptoms, including those specific to the ASD/GI population, we can begin to understand why some patients with ASD appear to respond less reliably to treatment than others with similar GI symptoms (7). Examining the downstream targets of miRNA differentially expressed in those with ASD and GI symptoms would likely contribute substantially to this understanding. Fortunately, with the ability to rapidly, and non-invasively measure miRNA in saliva (21), such information can readily be obtained from large populations. Development of such targeted approaches may provide opportunities for personalized treatment of gastrointestinal symptomatology, and lead to down-stream improvement in related behaviors (7, 10, 11), by impacting anxiety, mood, sleep, and attention (12–16).

This study harnesses, to our knowledge, the largest sample of the salivary micro-transcriptome in ASD. Its inclusion of children with non-ASD developmental delay as part of the control group is a relative strength. Inclusion of participants from multiple geographic sites also promotes generalizability of the findings. However, there are several limitations which should be noted. First, GI disturbances were identified through parent report and review of medical records but were not specifically assessed by physicians as part of the study. Second, we acknowledge that “GI disturbance” is a somewhat artificial distinction that groups together loosely related pathology that occurs in the GI tract. Important physiologic differences exist between conditions such as constipation and reflux, and these underlying biologic differences may have served to enhance false negative findings in the initial analyses. For this reason, we performed secondary analyses of the GI sub-phenotypes. However, this approach also has a trade-off of reducing the study's substantial sample size. Third, there are several pre-analytic factors which may potentially confound this study's findings, including batch effects and sample collection factors. We note that all samples were run on the same sequencing machine, using the same library preparation procedure, performed by the same laboratory technician. Although this analysis did not control for sample collection parameters, such as collection time, prandial status, or prior tooth-brushing, we have previously assessed the impact of many of these factors on the saliva microtranscriptome (21). We note that none of the microbial features and very few of the miRNA features identified in this study have demonstrated relationships with pre-analytic factors.

We recognize that the *salivary* transcriptome serves as a proxy for the primary pathologic site of most GI disturbances, the lower GI tract. However, several studies have reported significant overlap between saliva and stool micro-transcriptome features (41–43). Unlike the stool microbiome, we note that the saliva microbiome can be repeatedly sampled on demand and has shown resilience to antibiotic treatments (44). These characteristics make saliva an attractive source for sampling GI-related biology (particularly in patients with conditions such as reflux, eosinophilic esophagitis, or cyclic vomiting). Our previous work with parents of children with ASD has shown that they overwhelmingly prefer saliva as a clinical biofluid (45). Additionally, we note that association analyses between salivary transcriptome elements and ADOS scores rely solely on ASD and DD participants for whom these assessments were available. The lack of TD participants in this analysis could have impacted the findings. Finally, we must also consider the possibility that some of these markers may be caused by the downstream effects of the gastrointestinal symptoms or treatments, rather than serving a mechanistic role, but this would not diminish their potential use as biomarkers.

This is, to our knowledge, the first effort to examine the salivary RNA profile associated with GI symptoms in ASD, in a large population study. With the increased understanding of the critical importance of subtyping for meaningful precision medicine approaches in ASD (20), as well as the importance of GI symptomatology in behavioral issues in ASD (7, 10, 11), and the potential for mechanistic understanding through examination of the downstream targets of differentially regulated miRNAs, this is an important future direction of investigation. A subset of the micro-transcriptome features identified in this study displays relationships with treatment modality and are associated with autistic behaviors. The pathobiologic targets of these micro-transcriptome markers may serve as novel targets for individualized therapeutic interventions aimed at easing pain and behavioral difficulties seen in ASD-related GI disturbance.

## Data Availability Statement

The datasets presented in this article are not readily available due to the proprietary nature of the research. Requests to access the datasets should be directed to David Levitskiy, david.levitskiy@motionintel.com.

## Ethics Statement

The studies involving human participants were reviewed and approved by the Western Institutional Review Board (IRB #20180172). Written informed consent to participate in this study was provided by the participants' legal guardian/next of kin.

## Author Contributions

FM, RG-K, RS, DB, KS, and SH contributed to conception and design of the study. DL performed the statistical analysis. AC and PT contributed to data collection and formatting. DB, KS, and SH wrote the first draft of the manuscript. All authors contributed to manuscript revision, read, and approved the submitted version.

## Funding

This work was funded by an award from the National Institutes of Health to Quadrant Biosciences (R42MH111347).

## Conflict of Interest

DL and PT are paid employees of Quadrant Biosciences (QB). DB, KS, FM, and SH serve on the Scientific Advisory Board of QB. QB has licensed IP regarding the use of salivary transcripts for diagnosis of autism from the Penn State College of Medicine and SUNY Upstate Medical University, on which FM and SH are named as co-inventors. The remaining authors declare that the research was conducted in the absence of any commercial or financial relationships that could be construed as a potential conflict of interest.

## Publisher's Note

All claims expressed in this article are solely those of the authors and do not necessarily represent those of their affiliated organizations, or those of the publisher, the editors and the reviewers. Any product that may be evaluated in this article, or claim that may be made by its manufacturer, is not guaranteed or endorsed by the publisher.

## References

[1] Hicks SD Rajan AT Wagner KE Barns S Carpenter RL Middleton FA. Validation of a salivary RNA test for childhood autism spectrum disorder. Front Genet. (2018) 9:534. 10.3389/fgene.2018.00534 30473705 PMC6237842

[2] Huang Y Shen XJ Zou Q Wang SP Tang SM Zhang GZ. Biological functions of microRNAs: a review. J Physiol Biochem. (2011) 67:129–39. 10.1007/s13105-010-0050-6 20981514

[3] Hicks SD Middleton FA. A comparative review of microRNA expression patterns in Autism Spectrum disorder. Front Psychiatry. (2016) 7:176. 10.3389/fpsyt.2016.00176 27867363 PMC5095455

[4] Hicks SD Ignacio C Gentile K Middleton FA. Salivary miRNA profiles identify children with autism spectrum disorder, correlate with adaptive behavior, and implicate ASD candidate genes involved in neurodevelopment. BMC Pediatr. (2016) 16:52–52. 10.1186/s12887-016-0586-x 27105825 PMC4841962

[5] Hicks SD Carpenter RL Wagner KE Pauley R Barros M Tierney-Aves C. Saliva microRNA differentiates children with autism from peers with typical and atypical development. J Am Acad Child Adolesc Psychiatry. (2020) 59:296–308. 10.1016/j.jaac.2019.03.017 30926572 PMC6764899

[6] McElhanon BO McCracken C Karpen S Sharp WG. Gastrointestinal symptoms in autism spectrum disorder: a meta-analysis. Pediatrics. (2014) 133:872–83. 10.1542/peds.2013-3995 24777214

[7] Buie T Campbell DB Fuchs GJ Furuta GT Levy J VandeWater J. Evaluation, diagnosis, and treatment of gastrointestinal disorders in individuals with ASDs: a consensus report. Pediatrics. (2010) 125(Supplement 1):S1–18. 10.1542/peds.2009-1878C 20048083

[8] Peeters B Noens I Philips EM Kuppens S Benninga MA. Autism spectrum disorders in children with functional defecation disorders. J Pediatr. (2013) 163:873–8. 10.1016/j.jpeds.2013.02.028 23522863

[9] Sparks B Cooper J Hayes C Williams K. Constipation in children with autism spectrum disorder associated with increased emergency department visits and inpatient admissions. J Pediatr. (2018) 202:194–8. 10.1016/j.jpeds.2018.05.004 29866597

[10] Chaidez V Hansen RL Hertz-Picciotto I. Gastrointestinal problems in children with autism, developmental delays or typical development. J Autism Dev Disord. (2014) 44:1117–27. 10.1007/s10803-013-1973-x 24193577 PMC3981895

[11] Wasilewska J Klukowski M. Gastrointestinal symptoms and autism spectrum disorder: links and risks–a possible new overlap syndrome. Pediatr Health Med Ther. (2015) 6:153. 10.2147/PHMT.S85717 29388597 PMC5683266

[12] Doshi-Velez F Ge Y Kohane I. Comorbidity clusters in autism spectrum disorders: an electronic health record time-series analysis. Pediatrics. (2014) 133:e54–63. 10.1542/peds.2013-0819 24323995 PMC3876178

[13] Aldinger KA Lane CJ Veenstra-VanderWeele J Levitt P. Patterns of risk for multiple co-occurring medical conditions replicate across distinct cohorts of children with autism spectrum disorder. Autism Res. (2015) 8:771–81. 10.1002/aur.1492 26011086 PMC4736680

[14] Simonoff E Pickles A Charman T Chandler S Loucas T Baird G. Psychiatric disorders in children with autism spectrum disorders: prevalence, comorbidity, and associated factors in a population-derived sample. J Am Acad Child Adolesc Psychiatry. (2008) 47:921–9. 10.1097/CHI.0b013e318179964f 18645422

[15] Mazurek MO Vasa RA Kalb LG Kanne SM Rosenberg D Keefer A. Anxiety, sensory over-responsivity, and gastrointestinal problems in children with autism spectrum disorders. J Abnorm Child Psychol. (2013) 41:165–76. 10.1007/s10802-012-9668-x 22850932

[16] Ferguson BJ Dovgan K Takahashi N Beversdorf DQ. The relationship among gastrointestinal symptoms, problem behaviors, and internalizing symptoms in children and adolescents with autism spectrum disorder. Front Psychiatry. (2019) 10:194. 10.3389/fpsyt.2019.00194 31024357 PMC6465634

[17] Ferguson BJ Marler S Altstein LL Lee EB Mazurek MO McLaughlin A. Associations between cytokines, endocrine stress response, and gastrointestinal symptoms in autism spectrum disorder. Brain Behav Immun. (2016) 58:57–62. 10.1016/j.bbi.2016.05.009 27181180 PMC5526212

[18] Ferguson BJ Marler S Altstein LL Lee EB Akers J Sohl K. Psychophysiological associations with gastrointestinal symptomatology in autism spectrum disorder. Autism Res. (2017) 10:276–88. 10.1002/aur.1646 27321113 PMC5526214

[19] Furuta GT Williams K Kooros K Kaul A Panzer R Coury DL. Management of constipation in children and adolescents with autism spectrum disorders. Pediatrics. (2012) 130(Supplement 2):S98–105. 10.1542/peds.2012-0900H 23118260

[20] Beversdorf DQ Wang P Barnes G Weisskopf M Hardan A Hu V. Phenotyping, etiological factors, and biomarkers: toward precision medicine in autism spectrum disorder. J Dev Behav Pediatr. (2016) 7:659–73. 10.1097/DBP.0000000000000351 27676697 PMC5102276

[21] Hicks S. D. Khurana N Williams J Dowd Greene C Uhlig R Middleton FA. Diurnal oscillations in human salivary microRNA and microbial transcription: Implications for human health and disease. PLoS ONE. (2018) 13:e0198288. 10.1371/journal.pone.0198288 30020932 PMC6051604

[22] Langmead B Trapnell C Pop M Salzberg SL. Ultrafast and memory-efficient alignment of short DNA sequences to the human genome. Genome Biol. (2009) 10:R25. 10.1186/gb-2009-10-3-r25 19261174 PMC2690996

[23] Kozomara A Birgaoanu M Griffiths-Jones S. miRBase: from microRNA sequences to function. Nucleic Acids Res. (2019) 47:D155–62. 10.1093/nar/gky1141 30423142 PMC6323917

[24] Wang J Zhang P Lu Y Li Y Zheng Y Kan Y. piRBase: a comprehensive database of piRNA sequences. Nucleic Acids Res. (2019) 47:D175–80. 10.1093/nar/gky1043 30371818 PMC6323959

[25] O'Leary NA Wright MW Brister JR Ciufo S Haddad D McVeigh R. Reference sequence (RefSeq) database at NCBI: current status, taxonomic expansion, and functional annotation. Nucleic Acids Res. (2016) 44:D733–45. 10.1093/nar/gkv1189 26553804 PMC4702849

[26] Vlachos IS Zagganas K Paraskevopoulou MD Georgakilas G Karagkouni D Vergoulis T. DIANA-miRPath v3.0: deciphering microRNA function with experimental support. Nucleic Acids Res. (2015) 43:W460–6. 10.1093/nar/gkv403 25977294 PMC4489228

[27] Zhu Y Gu L Li Y Lin X Shen H Cui K. miR-148a inhibits colitis and colitis-associated tumorigenesis in mice. Cell Death Differ. (2017) 24:2199–209. 10.1038/cdd.2017.151 28960206 PMC5686357

[28] Hou Q Huang Y Zhang C Zhu S Li P Chen X et al. MicroRNA-200a targets cannabinoid receptor 1 and serotonin transporter to increase visceral Hyperalgesia in diarrhea-predominant irritable bowel syndrome rats. J Neurogastroenterol Motil. (2018) 24:656. 10.5056/jnm18037 30347941 PMC6175558

[29] Marler S Ferguson BJ Lee EB Peters B Williams KC McDonnell E. Brief report: whole blood serotonin levels and gastrointestinal symptoms in autism spectrum disroder. J Autism Dev Disord. (2016) 46:1124–30. 10.1007/s10803-015-2646-8 26527110 PMC4852703

[30] Campbell DB Buie TM Winter H Bauman M Sutcliffe JS. Distinct genetic risk based on association of MET in families with co-occurring autism and gastrointestinal conditions. Pediatrics. (2009) 123:1018–24. 10.1542/peds.2008-0819 19255034

[31] Ji LL Ye Y Nie PY Peng JB Fu CH Wang ZY. Dysregulation of miR-142 results in anxiety-like behaviors following single prolonged stress. Behav Brain Res. (2019) 365:157–63. 10.1016/j.bbr.2019.03.018 30857769

[32] Kim HK Tyryshkin K Elmi N Dharsee M Evans KR Good J. Plasma microRNA expression levels and their targeted pathways in patients with major depressive disorder who are responsive to duloxetine treatment. J Psychiatr Res. (2019) 110:38–44. 10.1016/j.jpsychires.2018.12.007 30580082

[33] Garofalo M Romano G Di Leva G Nuovo G Jeon YJ Ngankeu A. EGFR and MET receptor tyrosine kinase–altered microRNA expression induces tumorigenesis and gefitinib resistance in lung cancers. Nat Med. (2012) 18.1:74–82. 10.1038/nm.2577 22157681 PMC3467100

[34] Jyonouchi H Geng L Streck DL Toruner GA. Children with autism spectrum disorders symptoms exhibit distinct innate immune abnormalities and transcriptional profiles of peripheral blood (PB) monocytes. J Neuroimmunol. (2011) 238:73–80. 10.1016/j.jneuroim.2011.07.001 21803429

[35] Rose DR Yang H Carega M Angkustsiri K Van de Water J Ashwood P. T cell populations in children with autism spectrum disorder and co-morbid gastrointestinal symptoms. Brain Behav Immun Health. (2020) 2:100042. 10.1016/j.bbih.2020.100042 34589832 PMC8474588

[36] Luna RA Oezguen N Balderas M Venkatachalam A Runge JK Versalovic J. Distinct microbiome-neuroimmune signatures correlated with functional abdominal pain in children with autism spectrum disorder. Cell Mol Gastroenterol Hepatol. (2017) 3:218–30. 10.1016/j.jcmgh.2016.11.008 28275689 PMC5331780

[37] Kang DW Adams JB Gregory AC Borody T Chittick L Fasano A. Microbiota transfer therapy alters gut ecosystem and improves gastrointestinal and autism symptoms: an open-label study. Microbiome. (2017) 5:10. 10.1186/s40168-016-0225-7 28122648 PMC5264285

[38] Arnold LE Luna RA Williams K Chan J Parker RA Wu Q. Probiotics for gastrointestinal symptoms and quality of life in autism: a placebo-controlled pilot trial. J Child Adolesc Psychopharmacol. (2019) 29:659–69. 10.1089/cap.2018.0156 31478755 PMC7364307

[39] Li Q Johnston N Zheng X Wang H Zhang X Gao D. miR-28 modulates exhaustive differentiation of T cells through silencing programmed cell death-1 and regulating cytokine secretion. Oncotarget. (2016) 7:53735. 10.18632/oncotarget.10731 27447564 PMC5288217

[40] Bahmani L Baghi M Peymani M Javeri A Ghaedi K. MiR-141-3p and miR-200a-3p are involved in Th17 cell differentiation by negatively regulating RARB expression. Human Cell. (2021) 34:1375–87. 10.1007/s13577-021-00558-4 34086186

[41] Xun Z Zhang Q Xu T Chen N Chen F. Dysbiosis and ecotypes of the salivary microbiome associated with inflammatory bowel diseases and the assistance in diagnosis of diseases using oral bacterial profiles. Front Microbiol. (2018) 9:1136. 10.3389/fmicb.2018.01136 29899737 PMC5988890

[42] Segata N Haake SK Mannon P Lemon KP Waldron L Gevers D. Composition of the adult digestive tract bacterial microbiome based on seven mouth surfaces, tonsils, throat and stool samples. Genome Biol. (2012) 13:1–8. 10.1186/gb-2012-13-6-r42 22698087 PMC3446314

[43] Igaz I Igaz P. Diagnostic relevance of microRNAs in other body fluids including urine, feces, and saliva. Exp Suppl. (2015) 106:245–52. 10.1007/978-3-0348-0955-9_11 26608207

[44] Zaura E Brandt BW Teixeira de Mattos MJ Buijs MJ Caspers MP Rashid MU. Same exposure but two radically different responses to antibiotics: resilience of the salivary microbiome versus long-term microbial shifts in feces. MBio. (2015) 6:e01693–15. 10.1128/mBio.01693-15 26556275 PMC4659469

[45] Wagner KE McCormick JB Barns S Carney M Middleton FA Hicks SD. Parent perspectives towards genetic and epigenetic testing for autism spectrum disorder. J Autism Dev Disord. (2020) 50:3114–25. 10.1007/s10803-019-03990-6 30903561 PMC6755071

